# Next-Generation Allergen-Specific Immunotherapy for Food Allergy

**DOI:** 10.1007/s12016-025-09104-8

**Published:** 2025-10-22

**Authors:** Joyce Z. X. Lee, Jason K. C. Sit, Nicki Y. H. Leung, Ka Hou Chu, Patrick S. C. Leung, Ting Fan Leung, Christine Y. Y. Wai

**Affiliations:** 1 Department of Paediatrics, The Chinese University of Hong Kong, Hong Kong SAR, China; 2 School of Life Sciences, The Chinese University of Hong Kong, Hong Kong SAR, China; 3 Division of Rheumatology, Allergy and Clinical Immunology, University of California, Davis, CA USA; 4 Hong Kong Hub of Paediatric Excellence, The Chinese University of Hong Kong, Hong Kong SAR, China

**Keywords:** Allergen-specific Immunotherapy, Hypoallergen, Nanoparticl, Adjuvant, Immunomodulator

## Abstract

Allergen-specific immunotherapy (AIT) is currently the only disease-modifying treatment for food allergies. The most extensively studied form of AIT is oral immunotherapy, in which an increasing dose of specific food allergen is gradually introduced to allergic patients for immune system “re-education.” It has been demonstrated to effectively achieve desensitization, raising the threshold for inducing allergic reactions after allergen ingestion. However, lengthy dosing schedules and the occurrence of severe adverse events have impeded the adoption and compliance of oral immunotherapy. In recent years, extensive efforts in developing novel platforms have been directed to heighten the immunogenicity and lower the allergenicity of AIT, in hopes of increasing its efficacy and safety. Certain vaccine candidates have been investigated in preclinical and clinical trials. In this review, we aim to summarize the state-of-the-art technology of next-generation AIT vaccines for food allergy and explore research gaps in the field that warrant further investigation. We adopted a ‘Cargo-Truck-Lubricant’ analogy to illustrate the components of AIT, corresponding to modified allergens, carriers delivering the allergens, and the immunomodulators fostering the delivery. While most studies focused mainly on peanut allergy, novel AITs for other food allergies were still in preclinical stages. Future directions point towards optimization and the clinical translation of next-generation AIT vaccines to maximize the therapeutic outcome and minimize risks.

## Introduction

**Fig. 1:**
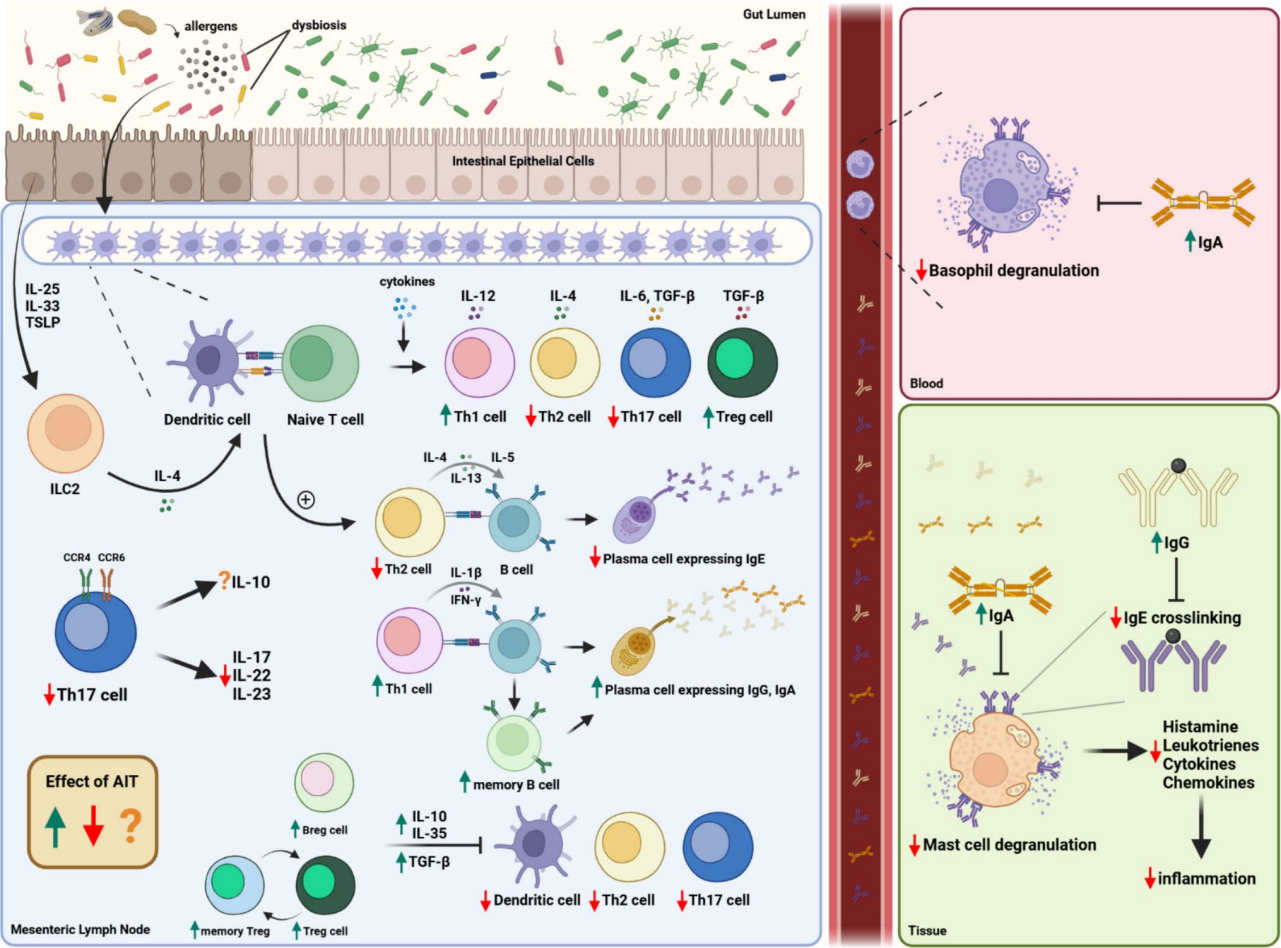
Mechanism of allergy and allergen-specific immunotherapy (AIT). In FA, upon antigen presentation by dendritic cells, naïve T cells differentiate into Th2 cells, which secrete cytokines including IL-4, IL-5, and IL-13 to stimulate the differentiation of B cells into IgE-producing plasma cells. IgE can bind to mast cells and basophils and become cross-linked upon allergen re-exposure, triggering cell degranulation to release inflammatory mediators. In AIT, specific food allergens are administered into FA patients to achieve tolerance to corresponding food. One of the targets of AIT is to re-establish the Th1/Th2 balance, in which IgE production would decrease, whereas IgA and IgG production would increase, inhibiting effector cell degranulation. While AIT reduces Th17 cell levels and their corresponding pro-inflammatory cytokines, AIT upregulates the production of regulatory T and B cells, which express immunosuppressive cytokines to mitigate inflammation. A plus sign (+) indicates that the corresponding pathway is enhanced in this context

The influence of food allergy (FA) worldwide is ever-increasing and greatly impacts the quality of life of food-allergic patients and their caregivers. In two epidemiological studies conducted by Gupta et al. in 2011 and 2018, the prevalence of FA among US children was estimated to be 8% [1, 2]. Notably, over 10% of 12-month-old infants were diagnosed with FA based on oral food challenge in Australia [3]. Although FA was generally thought to be more prevalent in industrialized areas, FA in developing countries has been shown to be increasingly prevalent. In Chongqing, China, the prevalence of challenge-proven FA in children less than 2 years of age rose from 3.5% in 1999 to 7.7% in 2009 [4]. Although certain types of FA such as egg and cow’s milk allergy can be outgrown during childhood [5, 6], FA such as shellfish and fish allergy can be long-lasting [7]. In FA, activation of type 2 T helper (Th2) cells and the release of cytokines (IL-4, IL-5, IL-13) leads to the differentiation of B cells into IgE-producing plasma cells (Fig. 1). The food-specific IgE binds to the surface of tissue-residing mast cells and circulating basophils, thus promoting the release of proinflammatory mediators such as histamine upon subsequent allergen exposure. Clinical manifestations range from mild skin rashes and respiratory symptoms to life-threatening systemic anaphylaxis. In the past, only symptom-relieving treatments such as antihistamines and epinephrine pens were available in the market.

Recently, biologics, for instance, anti-IgE antibodies, are under development for treating FA. Currently, Xolair® (Omalizumab) is the only humanized anti-IgE monoclonal antibody that received clinical approval from the US Food and Drug Administration (FDA) for the mitigation of allergic reactions by preventing the crosslinking of IgE and IgE receptors (FcεRI and FcεRII) [8, 9]. In the Phase 3 Omalizumab as Monotherapy and as Adjunct Therapy to Multi-Allergen Oral Immunotherapy in Food Allergic Children and Adults (OUtMATCH) trial, omalizumab was administered to patients with DBPCFC-confirmed peanut allergy and at least two other food allergies biweekly or monthly for 16 weeks. After treatment, 67% of peanut-allergic patients successfully consumed at least 600 mg of peanut protein during the food challenge, compared to 7% for those receiving placebo, leading to its approval for food allergies in > 1 year old patients by the US FDA in 2024 [10].

However, to genuinely improve the patients’ quality of life, disease-modifying treatments such as allergen-specific immunotherapy (AIT) are undoubtedly integral. In the context of AIT in FA, oral immunotherapy (OIT) is the most well-studied, which aims at re-educating the immune system of allergic patients by gradually introducing an increasing dose of specific allergens to them. The primary goal of OIT is to achieve desensitization, in which the threshold for triggering an allergic response upon allergen ingestion is raised, and ultimately sustained unresponsiveness (SU), offering patients long-term protection after treatment completion [11]. In a meta-analysis gathering data from 27 clinical trials, OIT has been demonstrated to efficaciously facilitate desensitization, though its effect to achieve SU was not confirmed [12]. The underlying mechanism of AITs involves re-establishing Th1/Th2 balance, inducing regulatory T and B cells, and promoting the synthesis of inhibitory IgG4 and IgA (Fig. 1). Despite the therapeutic effects of OIT, complying with a lengthy dosing schedule as long as 2–3 years is often considered difficult for patients. Moreover, OIT might cause severe adverse events including systemic anaphylaxis [13]. Therefore, in recent years, advancements have been developed to optimize treatment conditions, with the aim of improving patients’ compliance and reducing the occurrence of serious adverse events (SAEs).

**Fig. 2:**
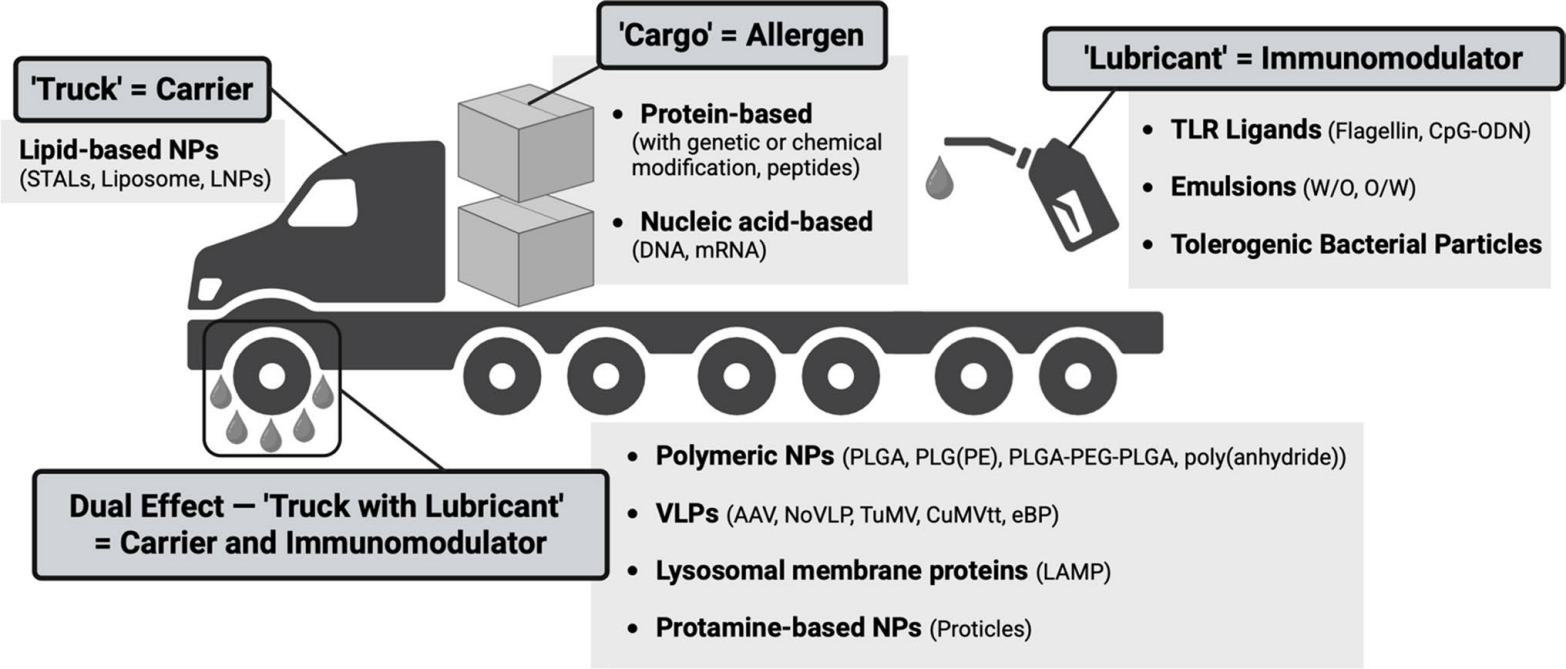
Proposed framework of AIT vaccines using the analogy of a “Cargo-Truck-Lubricant”. In AIT vaccine design, allergens or allergen derivatives act as the antigen or “cargo” to be delivered into the body, and they can be either protein-based or nucleic acid-based. Vaccine carriers like lipid-based NPs serve as “trucks” that transport the allergens into the body, while immunomodulators are a metaphor for “lubricants” that enhance or dampen the immunity, such as TLR ligands, emulsions, and tolerogenic bacterial particles. Moreover, polymeric NPs, VLPs, lysosomal membrane proteins, and protamine-based NPs have been studied in AIT for FA due to their dual effect as carriers with immunomodulatory properties, analogous to a “truck with lubricant”

Herein, we present an overview of the recent advancements in multiple facets of AIT including allergen modification, carrier strategies, and auxiliary addition. The AIT components will be discussed using a “Cargo-Truck-Lubricant” analogy: (1) “Cargo,” the allergens to be delivered, (2) “Truck,” the carriers delivering the allergens, (3) “Lubricant,” the immunomodulators facilitating delivery, and (4) the dual effect of “Truck with Lubricant” indicated as carriers with immunomodulatory properties (Fig. 2). While the “Cargo-Truck-Lubricant” analogy is a proposed framework for AIT, in a vaccine it is not necessary to include all three components.

## The “Cargo”—Allergen

Here, we define the “cargo” as the allergen used in the designer vaccine. In the past decades, efforts have focused on developing different protein-, peptide-, and nucleic acid-based “cargos” with the aim of improving the safety of AIT (Table 1).

**Table 1:** Preclinical development of next-generation “cargo” for AIT for food allergy

Allergen category	Type of FA	“Cargo”	Details	Testing Subject	ROA	Year [Ref]
**Protein-based**						
With genetic modifications	Shellfish	Met e 1	Met e 1 with 49 point mutations (MEM49) or with all IgE-binding epitopes deleted (MED171)	BALB/c mice	IP	2014 [14]
	Fish	Cyp c 1	Cyp c 1 with mutations in the two active calcium binding sites	BALB/c mice	SC	2007 [15] 2015 [16]
With chemical modifications (glycation)	Shellfish	Exo m 1, Pen a 1	Exo m 1 and Pen a 1 glycated with glucose, maltose, maltotriose, maltopentaose, or maltoheptaose	Human basophil cell model KU812, BALB/c mice	SC	2019 [17] 2021 [18]
Peptides	Peanut	Ara h 2	Peptide bearing IgE epitopes and a T cell epitope	New Zealand white rabbits And rat basophilic leukemia (RBL) RS-ATL8 cells	/	2017 [19]
	Shellfish	Met e 1	Peptide mixture bearing T cell epitopes of Met e 1	BALB/c mice	IG	2016 [20]
	Milk	Gly-m-Bd-30 K	Soy-derived-peptide bearing cross-reactive T and IgE epitopes with bovine caseins	BALB/c mice	IG	2021 [21]
	Peach	Pru p 3	Peptide bearing T-cell epitope of Pru p 3	BALB/c mice	SL	2019 [22] 2022 [23] 2023 [24]
	Shrimp	Arginine kinase (AK)	Peptide bearing T-cell epitope of AK	BALB/c mice	IP	2020 [25]
**Nucleic acid-based**						
DNA	Shellfish Tree nut	Shellfish and tree nut allergens	DNA encoding 5 shellfish allergens, 3 walnut allergens, or 3 pecan allergens	BALB/cJ, C3H/H3J, and CC027/GeniUnc mice	TC	2022 [26]
	Shellfish	Met e 1	DNA encoding Met e 1 with 49 point mutations (pMEM49) or with all IgE-binding epitopes deleted (pMED171)	BALB/c mice	ID	2014 [14] 2019 [27]
	Shellfish	Lit v 1	DNA encoding sequences of Lit v 1 and lysosomal-associated membrane protein (LAMP)	BALB/c mice	ID	2022 [28]
	Peanut	Ara h 1, 2, 3	DNA encoding sequences of the peanut allergens and LAMP	C3H/HeJ mice	ID	2015 [29]
mRNA	Peanut	Ara h 2	mRNA encoding a MHC-II targeting sequence and nonallergenic T cell epitopes of Ara h 2	C3H/HeJ mice	IV	2023 [30]

### Hypoallergens

Hypoallergens are derived from allergens that are deprived of their IgE-binding capability by either genetic or chemical modifications based on their IgE-binding epitopes. These epitopes can be predicted using in silico computational program, such as Bepipred Antibody Epitope Prediction [31], Emini Surface Accessibility Prediction [32], and Kolaskar & Tongaonkar Antigenicity model [33], or more precisely by probing the IgE binding regions through ELISA, dot-immunoblotting, and peptide microarray for linear epitopes, as well as nuclear magnetic resonance (NMR) spectroscopy to map the conformational epitopes [14, 34, 35]. Armed with this essential information, native allergens can be modified accordingly and tested for their in vitro and in vivo allergenicity. Our group has constructed two hypoallergenic variants of shrimp tropomyosin (TM) Met e 1 by introducing 49-point mutations (MEM49) or by deleting all nine IgE-binding epitopes (MED171). Intraperitoneal injections of MEM49 or MED171 were capable of inducing protective Met e 1-specific IgG_2a_ antibodies (equivalent to human IgG_4_) in mice intragastrically sensitized and challenged with recombinant TM [14]. A recombinant hypoallergenic parvalbumin with two mutations in the calcium-binding domain has been shown to exhibit a 95% reduction in IgE reactivity by dot blotting and western blotting and also induce IgG_1_ production in immunized mice [15, 16]. Subsequently, two phase 1/2 and phase 2 clinical trials (NCT02017626 and NCT02382718) were conducted to evaluate its safety and efficacy in the form of subcutaneous immunotherapy, although the results are yet to be published.

Glycation, an enzyme-independent reaction between proteins and reducing sugars, is one of the chemical means to produce hypoallergens. The addition of ribose, galacto-oligosaccharide, or chitosan-oligosaccharide to lysine-rich shrimp TM by Maillard reaction, in which the carbonyl group of the reducing sugars interacts with the ε-amino group of lysine in the protein, has been shown to reduce IgE-binding affinity of TM by altering its tertiary structure, with a decrease of α-helices and an increase of β-sheets, as monitored by far-UV circular dichroism [36]. Another group measured the allergenicity of TM glycated by saccharides of different molecular sizes in the human basophil cell model KU812. Lower levels of β-hexosaminidase, histamine, and IL-4 were found in the culture medium of IgE-sensitized KU812 upon the addition of glycated TMs when compared to unmodified TM, implying a lower degree of basophil degranulation and its hypoallergenic nature, except for maltose-glycated TM [17]. Zhang et al. furthered the investigation on a mouse model, in which glycated TM was administered subcutaneously twice per week for 6 weeks. Compared to TM treatment controls, glycated TM treated mice had a lower anaphylactic score, IgE level, proinflammatory cytokines including IL-4 and IL-13, but elevated production of IgG2a, anti-inflammatory mediators such as IL-10 and TGF-β, suggesting SCIT using glycated allergen as a safe and effective approach to treat shrimp allergy [18].

### Peptide-Based Immunotherapy

Peptides bearing T cell or IgE-binding epitopes are also emerging candidates in FA AIT. Short peptides lack the ability to crosslink IgE-sensitized basophils or mast cells, which can minimize degranulation and IgE-mediated allergic side effects. Anzengruber et al. developed a peptide vaccine, AH3a42, targeting peanut allergy. AH3a42 is an Ara h 2-derived 42-amino-acid-long peptide bearing three IgE epitopes and a T cell epitope. Immunization of rabbits with AH3a42 in conjunction with surface layer protein from *Lactobacillus buchneri *induced production of AH3a42-specific IgG, which was capable of inhibiting 13.4% of IgE from peanut-allergic children from binding to Ara h 2 in competitive inhibition ELISA [19]. In the same study, while the addition of natural Ara h 2 at 10 pg/ml induced the release of beta-hexosaminidase from rat basophil leukemia RS-ATL8 cells sensitized with serum from peanut allergic patients, the addition of AH3a42 up to 100 ng/ml was unable to prompt beta-hexosaminidase release, indicating minimal mast cell degranulation induced by AH3a42 [19].

We have investigated the effect of T cell epitope immunotherapy in a mouse model of shrimp allergy using a peptide mixture comprising six individual 20-mers of different T cell epitopes that was intragastrically delivered to TM sensitized mice two times a week for 4 weeks. T cell epitope-treated mice showed lower symptom scores and recovered from intestinal inflammation, as evidenced by a significantly lower number of mucus-secreting goblet cells and eosinophils in the jejunum when compared with TM-treated control mice [20]. Another study reported that mice pre-treated with soy-derived 15-amino-acid-long peptide (P3) containing cross-reactive B and T cell epitopes of bovine casein, the major allergen in cow’s milk, had significantly lower clinical scores of allergic responses compared to mice sham-treated with PBS. It was also noted that a significantly lower level of IgE and a higher level of IgG2a without statistical significance were observed in the P3-treated group compared to PBS-treated mice [21].

Rodriguez et al. investigated the efficacy of sublingual administration of a glycodendropeptide (D1ManPrup3) comprising mannose dendron and T cell epitopes of Pru p 3, the major peach allergen, in a mouse model of peach allergy [22–24]. When bound to antigens, mannose dendron has been shown to facilitate antigen uptake by dendritic cells (DCs), which express mannose-binding receptors [37]. After D1ManPrup3 treatment, decreased antigen-specific IgE (sIgE) and increased IL-10 levels were observed and persisted for at least 5 weeks [22]. Moreover, bisulphite sequencing of regulatory T cells (Tregs) and DCs revealed distinct DNA methylation changes in mice treated with 2 nM or 5 nM D1ManPrup3 [23, 24], the former of which was shown to induce transient desensitization, whereas the latter of which was capable of inducing long-term tolerance to allergens [22]. Notably, Foxp3 was exclusively hypomethylated in the tolerant group [23, 24]. Another group investigated the efficacy of a previously reported T cell epitope of arginine kinase (AK) on treating shrimp allergy in a murine model [25, 38]. A 20-amino-acid-long peptide spanning the T cell epitope of AK was synthesized and co-encapsulated with the TLR9 agonist CpG-ODN using PLGA nanoparticles (CpG-AKp NPs). A lower diarrhea score, suppressed Th2 cytokine production, and increased Foxp3 and IL-10 levels were observed after CpG-AKp NPs treatment [25].

Protein vaccines mentioned above are often produced through recombinant technology, in which fusion allergens are expressed in and purified from host systems such as bacteria and insect cells. However, it is inevitable that minute amounts of host proteins are present in the purified vaccine. Moreover, protein misfolding can occur during the expression process, and the absence of crucial post-translational modifications is not uncommon due to differences in host systems. Taken together, these result in a higher risk of side effects and reduced efficacy of protein-based vaccines.

### Nucleic Acid-Based AIT

Nucleic acid-based vaccine, including DNA and mRNA, is a promising strategy for AIT. Depending on the route of administration, myocytes or keratinocytes are the first line of cells encountering the DNA vaccine, which will be transcribed into mRNA and then translated into protein. With this approach, the inclusion of exogenous proteins from other host systems could be avoided, circumventing the limitations of protein-based vaccines. Taken up by DCs, the allergens are presented to T cells in the lymph node and prompt subsequent immune responses. A recent study has demonstrated the superiority of transcutaneous DNA vaccine delivery with Gene Gun over intramuscular injection [26]. DNA vaccines encoding shellfish or walnut allergens were tested on shrimp or walnut extract-sensitized BALB/c, C3H/HeJ, and CC027/GeniUnc mice. Increased IgG levels were observed in all three strains of vaccinated mice, with the effect boosted by IL-12, a strong Th1-skewing cytokine, in certain strains [26].

Our group has tested the efficacy of a DNA vaccine encoding hypoallergens of shrimp TM (pMEM49 and pMED171) as a therapeutic approach to treat shellfish allergy in a mouse model [14, 27]. Both intradermal treatment with pMEM49 and pMED171 markedly relieved allergic symptoms and reduced IgE and Th2 cytokine levels in TM-sensitized and challenged mice, the latter of which particularly enhanced responses of gut-homing Treg cells, as evidenced by Foxp3+cell accumulation in the Peyer’s patches and upregulated expression of gut-tropic integrin α4β7 in pMED171-treated mice [27]. Kubo et al. developed a DNA vaccine encoding shrimp TM and a lysosomal protein LAMP, which can directly be shuttled to the lysosomal compartment after protein synthesis [28]. When intradermally delivered into mice, significant reductions of anaphylaxis score and heightened TM-specific IgG2a level were evident in vaccinated mice compared to PBS-treated mice [28].

Li et al. developed a peanut allergy DNA vaccine, ASP0892, which is a DNA construct comprising sequences of peanut allergens Ara h1–3 and LAMP [29]. Based on promising results obtained from the preclinical trial for intradermal injections of ASP0892 in mice [29], Ferslew et al. extended the investigation to two phase 1 clinical trials, evaluating the safety and efficacy of ASP0892 (Table 3). Intradermal or intramuscular administration of ASP0892 in peanut-allergic patients with mild to moderate symptoms only led to a slight increase in IgG and IgG4 levels, with no statistical significance when compared with the placebo group [39]. Moreover, while ASP0892 was well tolerated by the recipients, no discernible improvements in clinical outcomes were observed, which could possibly be attributed to the low bioavailability and immunogenicity of ASP0892 [39].

Similar to DNA vaccine, mRNA vaccine, best known for COVID-19, utilizes cellular machinery to produce encoded allergens without requiring prior translocation to the nucleus for transcription and thus eliminates the concern of DNA incorporation into the genome. A large-scale phase 2/3 clinical trial of BNT162b2, a mRNA vaccine encoding the spike protein of severe acute respiratory syndrome coronavirus 2 (SARS-CoV-2), has shown that it is efficacious in preventing COVID-19 in 95% of participants over 16 years of age [40]. Although studies of mRNA vaccines are mainly focused on autoimmune diseases such as multiple sclerosis [41], there is one particular study investigating the effect of Ara h 2-encoded mRNA construct encapsulated by lipid nanoparticle on peanut allergy as prophylactic and therapeutic approaches [30]. This vaccine is designed based on the ability of liver sinusoidal endothelial cells (LSECs) acting as tolerogenic antigen-presenting cells (APCs), leading to the induction of Treg response. In this respect, the ability to facilitate liver biodistribution was achieved by surface decoration with lipid-anchored mannose ligand, and the encapsulated mRNA was designed to encode for the MHC II binding epitopes of the Ara h 2 [30]. Intravenous injections of mRNA encoding T-cell epitopes of Ara h 2 before or after anaphylaxis induction in C3H/HeJ mice prevented the onset of hypothermia or suppressed hypothermic response respectively, with concomitant induction of IL-10-producing splenic Tregs [30].

## “Truck” and “Lubricant”—Adjuvants for AIT

Other than the above AIT strategies, which focused on the allergens themselves, recent studies on AIT for FA have explored the use of novel adjuvants in conjunction with the modified or native allergens in vaccine formulation. Peptides derived from the allergens often exhibit low immunogenicity [42]; the incorporation of immunogenic adjuvants in FA AIT formulation can thus promote their immunogenicity to maximize their therapeutic effects. As a form of tolerogenic therapy itself, a few studies of FA AIT also investigated the potential of tolerogenic adjuvants with tolerogenic properties to enhance tolerogenic immune responses and redirect the Th1/Th2 immune balance. In the following sections, we discuss the application of novel adjuvants in AIT for FA, with the adjuvants categorized as either a carrier, an immunomodulator, or both a carrier and an immunomodulator. This classification is illustrated using the “cargo-truck-lubricant” analogy, where the carrier is metaphorically represented as a “truck” and the immunomodulator as “lubricant.”

## The “Truck”—Carrier

A carrier can be described as a “truck” that delivers the antigens into the body. While most adjuvants that serve as carriers have intrinsic immunogenic properties, one classic example of a carrier without such properties is a lipid-based nanoparticle.

### Lipid-Based Nanoparticles (LNPs)

LNPs are nanocarriers consisting of lipids that act as carriers for vaccine antigens. One type of lipid-based nanoparticle investigated in FA AIT is a liposome [43]. Liposomes are biodegradable, non-immunogenic spherical vesicles composed of natural or synthetic bilayer phospholipids, making them effective carriers for both hydrophilic and hydrophobic antigens, such as proteins and nucleic acids. In vivo, liposomes exert a depot effect that provides a prolonged, controlled release of the antigen. However, due to the lack of intrinsic immunogenicity, liposomes are often administered with other adjuvants or immunomodulators in the vaccine formulation [44].

Paulson et al. leveraged liposomes to develop Siglec-engaging tolerance-inducing antigenic liposomes (STALs) by displaying a ligand of Siglec-2 on the liposomal surface for the treatment of peanut allergy [45, 46]. Siglec-2, also known as CD22, is an inhibitory receptor from the Siglecs (sialic acid–binding immunoglobulin-type lectins) family mainly expressed on B cells, which negatively regulates B cell receptor signalling (Table 2). Thus, the rationale for administering Siglec-2 ligands alongside allergens is to prevent the activation of allergen-specific B cells, resulting in reduced allergen-specific IgE production. By co-displaying the major peanut allergen, Ara h 2, on STALs, the ability to sensitize naïve mice was significantly reduced as compared to the Ara h 2 displayed on liposomes [45]. A subsequent study administering STALs co-displaying peanut allergens (Ara h 1, Ara h 2, or Ara h 3) prevented anaphylaxis-associated hypothermia in peanut allergic mice with concomitant reduction in allergen-specific IgE and IgG1 [46]. Peanut-sensitized mice receiving two doses of immunizations showed a superior antibody response compared to a single dose and demonstrated sustained unresponsiveness to peanut extract for at least 3 months after the first challenge. Moreover, an egg allergy vaccine was constructed by the same group leveraging a similar approach of co-displaying liposomes with ovalbumin and a synthetic glycan ligand for Siglec-8, an inhibitory receptor expressed on mast cells and eosinophils [47]. This vaccine profoundly suppressed IgE-mediated mast cell and basophil degranulation in vitro. Since Siglec-8 is uniquely expressed on human mast cells and eosinophils [48], the therapeutic potential of the vaccine was tested in vivo on transgenic mice with mast cells expressing Siglec-8. Upon ovalbumin sensitization, immunization with the vaccine protected transgenic mice but not mice without Siglec-8 expression, demonstrating Siglec-8 dependent mast cell desensitization [47].

**Table 2:** Preclinical development of next-generation “truck” and “lubricant” in AIT for food allergy

Adjuvant type	Adjuvants	Type of FA	“Cargo”	Details	Testing subject	ROA	Year [Ref]
**Carrier, the “truck”**							
Lipid-based NP	STALs (120 ± 20 nm)	Peanut	Ara h 2	Siglec-engaging tolerance-inducing antigenic liposomes (STALs) co-displaying Ara h 2 (genetic fusion)	BALB/c mice	Oral	2017 [45]
			Ara h 1, Ara h 2, Ara h 3	STALs co-displaying Ara h 1, Ara h 2, or Ara h 3 (genetic fusion)	BALB/cJ and C57BL/6 mice	IV	2022 [46]
	Liposome	Egg	Ovalbumin	Liposome displaying OVA with synthetic glycan ligand for Siglec-8 (genetic fusion)	Siglec-8 transgenic mice, mouse bone marrow-derived mast cells (mBMMCs) and RBL cells	IV	2021 [47]
	LNPs (150 nm)	Peanut	mRNA encoding Ara h 2 T cell epitope 4	mRNA encapsulated in LNPs with mannose for surface decoration	C3H/HeJ mice	IV	2023 [30]
**Carrier and immunomodulator, the “truck with lubricant”**							
Polymeric NPs	PLGA	Peanut	Peanut extract	CpG-coated PLGA containing peanut extract (214 ± 39 nm)	C3H/HeJ mice	Oral	2016 [49]
		Shrimp	T cell epitope of arginine kinase	CpG-coated PLGA containing arginine kinase T cell epitope (195.5 nm)	BALB/c mice	IP	2020 [25]
		Milk	β-lactoglobulin peptide	BLG and CpG-ODN co-encapsulated in PLGA (280 nm)	C3H/HeOuJ mice	Oral	2018 [50] 2022 [51]; 2023 [52]
	PLG(PE) (500 nm)	Peanut	Peanut extract	Peanut extract either encapsulated in PLG(PE) or displayed on the surface of PLG(PE) by carbodiimide-mediated coupling	C3H/HeJ and BALB/c mice	IV	2022 [53]
		Egg	Ovalbumin	Ovalbumin encapsulated in PLG(PE)	BALB/c mice	IV, IMuc	2024 [54]
		Alpha gal	αGal glycoprotein	αGal glycoprotein encapsulated in PLG(PE)	C57BL/6 mice	IV	2024 [55]
	PLGA-PEG-PLGA (105 ± 54 nm)	Egg	Hen egg lysozyme (HEL)	HEL-loaded PLGA-PEG-PLGA	BALB/c mice	PC	2024 [56]
	Poly(anhydride) (172–201 nm)	Peanut	Peanut extract	Peanut extract encapsulated in poly(anhydride) NPs	C57BL/6 mice	Oral	2014 [57]; 2017 [58]
		Cashew nut	Cashew nut extract	Cashew nut extract encapsulated in poly(anhydride) NPs	BALB/c mice, Macrophages RAW 64.7	Oral	2018 [59]
VLP	AAV (25 nm)	Egg	Ovalbumin-derived B-cell epitope	AAVLP displaying B cell epitope of OVA (genetic fusion) ad mixed with alum or Montanide® ISA 51 adjuvant	BALB/c mice, RBL cells	SC	2014 [60]
	NoVLP (23–40 nm)	Peach	Pru p 3	NoVLP displaying Pru p 3 (genetic fusion)	BALB/c mice	Oral; SC	2020 [61]
	TuMV (400 nm)			TuMV displaying Pru p 3 (genetic fusion)	C3H mice	SL	2022 [62]
	CuMVtt (36 nm)	Peanut	Roasted peanut (Ara R), Ara h 1, Ara h 2	CuMVtt displaying Ara R, Ara h1, or Ara h 2 (chemical coupling)	BALB/c mice	SC	2020 [63]
			Ara h 2	Mosaic VLP with two proteins: CuMVtt and CuMVtt displaying Ara h 2 (genetic fusion)	BALB/c mice	SC	2023 [64]
	Plant-based enveloped bioparticles (eBP) (150 nm)	Peanut	Ara h 2	Ara h 2 displayed on surface of plant eBP (genetic fusion)	mBMMCs, RBL cells, and monocyte-derived dendritic cells (moDCs) cocultured with allogeneic naïve CD4 T cells	-	2023 Aug [65]; 2023 Nov [66]
Lysosomal membrane proteins	LAMP	Peanut	Ara h 1, 2, 3	DNA encoding sequences of the peanut allergens and lysosomal-associated membrane protein (LAMP)	C3H/HeJ mice	ID/IM	2015 [29], 2024 [39]
		Shellfish	Lit v 1	DNA encoding sequences of Lit v 1 and LAMP	BALB/c mice	ID	2022 [28]
Protamine-based NPs	Proticles (600–1300 nm)	Peanut	Ara h 2	Non-covalent attachment of Ara h 2 to preformed Proticles by ionic interactions	BALB/c mice; RBL cells	SC	2013 [67]
**Immunomodulator, the “lubricant”**							
TLR ligands	Flagellin (TLR-5 agonist)	Egg	OVA	rFlaA genetically fused to rOVA	BALB/c mice, moDCs-T cell co-culture	IP	2010 [68]; 2011 [69]
		Egg	OVA	Flagellin ad mixed with ovalbumin	BALB/c mice	Oral	2022 [70]
	CpG-ODN (TLR-9 agonist)	Shrimp	T cell epitope of arginine kinase	CpG-coated PLGA containing arginine kinase T cell epitope (195.5 nm)	BALB/c	IP	2020 [25]
		Milk	β-lactoglobulin peptide	BLG and CpG-ODN co-encapsulated in PLGA (280 nm)	C3H/HeOuJ mice	Oral	2022 [52]
		Peach	Pru p 3 T-cell epitope	Pru p 3 peptide bound to mannose dendrons with CpG-ODN	BALB/c mice	SL	2019 [22]; 2022 [23]; 2023 [24]
		Peanut	Peanut extract	CpG-coated PLGA containing peanut extract (214 ± 39 nm)	C3H/HeJ mice	Oral	2016 [49]
			Ara h 2	Non-covalent attachment of Ara h 2 to preformed Proticles by ionic interactions	BALB/c mice; RBL cells	SC	2013 [67]
			Peanut powder	Mixture of peanut powder, CpG-ODN and VD3 packaged in a powder-laden, dissolvable microneedle array (PLD-MNA)	BALB/c and C57BL/6 mice	EP	2020 [71]
Emulsions	Water-in-oil (W/O) emulsions	Egg	Ovalbumin-derived B-cell epitope	AAVLP displaying B cell epitope of OVA (genetic fusion) ad mixed with alum or Montanide® ISA 51 adjuvant	BALB/c mice, RBL cells	SC	2014 [60]
	Oil-in-water (O/W) emulsions (400–500 nm)	Peanut	Peanut extract	NE ad mixed with peanut extract	C3H/HeJ and BALB/c mice	IN	2018 [72]; 2020 [73]
		Milk	Casein	NE ad mixed with casein	BALB/c mice	IN	2020 [74]
		Egg & peanut	Ovalbumin and/or peanut extract	NE ad mixed with ovalbumin and/or peanut extract	BALB/c mice	IN	2021 [75]
Tolerogenic bacterial particles	TBP from Lactococcus lactis probiotic	Egg	Ovalbumin	TBPs ad mixed with ovalbumin	Piglets	TD	2021 [76]
		Tree nut	Walnut extract	TBPs ad mixed with walnut extract	PBMC of tree-nut allergic patients	-	2022 [77]

In addition to the recent liver-targeting liposome-encapsulated mRNA vaccine for peanut AIT described earlier [30], these studies have highlighted the advantages of using liposomes as a carrier to direct pathway-targeted effects for FA AITs. Despite the promising results, none of the LNP-based vaccines for FA have progressed to clinical trials to our knowledge. Considering previous clinical trials of LNPs for other health disorders, it is crucial to consider their limitations, such as stability, complex manufacturing process, and safety concerns for humans [78, 79].

## The “Truck with Lubricant”—Carrier and Immunomodulator

A carrier with immunomodulatory properties can be described as a “truck with lubricant” that aids in the delivery of antigens into the body while simultaneously modulating the immune system. Except for lipid-based NPs, all carriers studied as novel adjuvants now in FA AIT are immunostimulants, namely polymeric NPs, virus-like particles, lysosomal-associated membrane proteins, and protamine-based NPs (Table 2).

### Polymeric NPs

Polymeric nanoparticles are 1–1000 nm nanostructures made from polymers that serve as carriers for antigens via encapsulation or surface adsorption [80]. Poly(lactic-co-glycolic) acid (PLGA) is a biodegradable synthetic polymer and is the most well-studied polymeric NPs in AIT [79]. Its applicability in FA treatment, including peanut [49, 53], shrimp [25], milk [50–52], egg [54], and alpha-gal [55] allergy, has been widely investigated. In 2016, Sampson et al. reported the efficacy of cytosine-phosphorothioate-guanine (CpG)-coated PLGA encapsulated with peanut extract in protecting peanut-sensitized mice from anaphylaxis, characterized by decreased peanut-specific IgE, IgG1, IL-4, IL-5, and IL-13 cytokines with concomitant increased peanut-specific IgG2a and IFN-γ cytokines [49]. The ability of PLGA to deliver less immunogenic molecules, such as short peptides, was demonstrated by a study that encapsulated the 20-amino-acid T-cell epitope of the shrimp allergen AK in the CpG-coated PLGA [25]. Sensitized mice were protected from anaphylaxis after six daily intraperitoneal immunizations, denoted by lower AK-specific IgE, IgG1, IL-4, and IL-13 cytokines and the concomitant induction of AK-specific IgG2a, IFNγ, and IL-10 cytokines. Willemsen et al. from the Netherlands also designed a similar vaccine formulation using PLGA co-encapsulated with CpG-ODN and T-cell epitopes of β-lactoglobulin (BLG), a cow’s milk allergen [50]. Six daily oral intakes of the co-encapsulated PLGA vaccine (40 µg per peptide) were claimed to partially prevent the development of whey-induced skin allergic reactions in mice, in which the prophylactic treatment of BLG-peptide PLGA NPs resulted in significantly lower acute allergic skin response in sensitized mice upon challenge than the PLGA control group but was not significantly lower than the PBS control group. Subsequent studies delineated a dose-dependent prophylactic effect of the BLG-peptides PLGA NPs, in which a high dose of vaccine (80 µg per peptide) better protected allergy development in mice against whole whey protein [51]. As compared to encapsulation with either CpG or BLG, the co-encapsulation of CpG and BLG peptide in PLGA was crucial in inducing tolerogenic splenic DCs, leading to lowered Th2 response and increased Treg/Th2 and Th1/Th2 ratios [52].

Despite the promising preclinical studies, the degradation and acid by-products of PLGA could potentially affect the stability of encapsulated antigens [78]. To circumvent this issue, PLGA can be coated with stabilizers such as polyethylene glycol (PEG). For instance, O’Konek et al. developed PLG(PEG) NPs with peanut extract encapsulated or surface displayed [53]. In addition to its prophylactic and therapeutic efficacies on peanut-sensitized mice, the study also demonstrated the ability of the NPs to inhibit delayed-type hypersensitivity. The promising results led to the initiation of a phase 1 clinical trial (NCT05250856) in peanut-allergic patients, but the trial was prematurely terminated due to unknown reasons (Table 3). The research group also extended the application of the NPs for the treatment of egg [54] and alpha-gal [55] allergies through the encapsulation of ovalbumin and α-Gal glycoprotein, respectively, which similarly suppressed Th2 response and mast cell degranulation while inducing tolerogenic immune responses. Sakurai R. et al., who recently explored the efficacy of percutaneous immunotherapy using hen egg lysozyme (HEL)-loaded PLGA-PEG-PLGA NPs, showed a high retention rate in the skin with increased sIgG1 and decreased sIgE [56].

**Table 3:** Clinical development of next-generation AIT for food allergy

Type of FA	Clinical trial ID	Adjuvants/product name	Target age group	ROA	Current state of clinical investigation
Fish	NCT02017626	mCyp c 1, a modified hypoallergenic parvalbumin with two mutations in the calcium-binding domain	18–65 y/o (fish allergic patients)	SC	Phase 1/2, completed Start: Aug 2013 End: Oct 2014 = Results unpublished
Fish	NCT02382718	mCyp c 1, a modified hypoallergenic parvalbumin with two mutations in the calcium-binding domain	18–65 y/o (fish allergic patients)	SC	Phase 2, completed Start: Oct 2015 End: Apr 2017 = Results unpublished
Peanut	NCT05250856	CNP-201 comprised of purified peanut extract (PPE) drug substance dispersed within a negatively charged polymer matrix of poly (lactic-co-glycolic acid) (PLGA) particles at a target concentration of ~ 5 μg of PPE per mg of PLGA	16–55 y/o (peanut allergic patients)	IV	Phase 1, terminated Start: March 2023 Expected end: Dec 2023
Peanut	NCT05476497	Mosaic VLP with CuMVtt and CuMVtt displaying Ara h 2	18–50 y/o (healthy and peanut allergic subjects)	SC	Phase 1 (PROTECT), ongoing Start: Oct 2022 Expected end: Oct 2025
Peanut	NCT02851277	ASP0892 (ARA LAMP Vax), a single multivalent peanut (Ara h 1, h2, h3) lysosomal associated membrane protein DNA plasmid vaccine	18–55 y/o (peanut allergic patients)	ID/IM	Phase 1, completed Start: Dec 2016 End: Dec 2018= Trial results: [39]
Peanut	NCT03755713	ASP0892 (ARA LAMP Vax), a single multivalent peanut (Ara h1, h2, h3) lysosomal associated membrane protein DNA plasmid vaccine	12–17 y/o (peanut allergic patients)	ID	Phase 1, completed Start: Mar 2019 End: Oct 2021 = Trial results: [39]

*Route of administration (ROA): *IV*, intravenous; *IP*, intraperitoneal; *IMuc*, intramucosal; *IN*, intranasal; *SL*, sublingual; *PC*, percutaneous; *EP*, epicutaneous; *TD,* transdermal

Poly(anhydride) is another biodegradable synthetic polymer whose degradation products are non-cytotoxic and less acidic than PLGA [78]. Poly(anhydride) is less explored in FA AIT compared to PLGA. A specific type of poly(anhydride), known as poly(methyl vinyl ether-co-maleic anhydride) (PVMA), was found to be a potential candidate as an oral vaccine adjuvant due to its strong bioadhesive affinity with gut mucosa components, leading to enhanced gut retention of the vaccine [78]. Gamazo et al. incorporated such poly(anhydride) to encapsulate peanut extract and stored the resulting NPs either as spray-dried or lyophilized form [57]. While oral immunization with 25 mg of the resuspended NPs from both formulations induced low sIgE and a Th1/Th2 balanced immunity, the spray-dry formulation was chosen for downstream studies due to its superior stability and Th1-promoting effect. A subsequent study confirmed the ability of the oral AIT to protect mice from peanut-induced anaphylaxis, as characterized by the reduction of total IgE, allergic symptoms, and mortality rate, with a concomitant increase in IFN-γ, IL-17, and IL-10 [58]. Another study on cashew nut allergy also demonstrated the therapeutic efficacy of poly(anhydride) encapsulated with cashew nut extract in inducing strong Th1 and Treg responses in peanut-allergic mice after oral immunization [59].

### Virus-Like Particles

Virus-like particles (VLPs) are biodegradable nanoparticles consisting of capsid proteins of viruses without viral genomic materials ranging from 20 to 200 nm in size [81]. The size of VLPs facilitates efficient uptake by APCs and effective lymphatic drainage, leading to potent activation of T and B cells. Moreover, the geometric symmetry of VLPs, which mimics the natural structure of viruses, not only enhances particle stability but also enables a repetitive array of antigen display on VLPs, promoting T cell-independent B cell activation and resulting in strong antibody responses. In addition, subunits of the VLPs can be expressed in a heterologous expression system such as bacteria, yeast, insects, plants, and mammalian cells, which could then self-assemble into the morphology of a native virus, making VLPs a safe, scalable, and immunogenic carrier for foreign antigens [82].

In the context of AIT, VLPs carrying Fc fragments of IgE that induce long-lasting anti-IgE IgG antibody to protect immunized mice from anaphylaxis are of immense interest [83]. Yet, VLPs are more often designed to display allergens to reduce their allergenicity compared to free allergens due to the distant spacing of epitopes on VLPs, which prohibits IgE cross-linking, making them potent candidates for AIT [84].

To the extent of this review, there have been five different types of VLPs designed for FA AIT. The earliest application of VLPs for FA AIT was in 2014 by Jensen-Jarolim et al. who leveraged adeno-associated virus (AAV) displaying the B-cell epitope of ovalbumin for the treatment of egg allergy [60]. Norovirus-like particles (NoVLPs) were also designed by Tome-Amat et al. as FA vaccine candidates due to their high immunogenicity with Th1-promoting ability and their versatility to conjugate large protein inserts in the three external loops [61]. The NoVLPs were successfully displayed with Pru p 3 peach allergens on their surface via genetic fusion, while the investigation of the vaccine’s immunogenicity and therapeutic efficacies was proposed but not reported. The same group also designed another type of VLPs derived from turnip mosaic virus (TuMV), and the resulting VLP construct displaying Pru p 3 was investigated for the treatment of peach allergy [62]. In 2020, Bachmann et al. proposed the use of cucumber mosaic virus including tetanus toxin epitopes (CuMVtt)–derived VLPs as the adjuvant for AIT of peanut allergy [63, 64]. This vaccine candidate is the first and currently the only VLP-based vaccine translated to clinical trial, which will be discussed in detail below. Lastly, a plant-based enveloped bioparticle (eBP) was recently developed by van Ree R. et al. which comprised the transmembrane domain and the cytosolic tail domain of influenza virus hemagglutinin [85]. The resulting eBP is distinctive from the four types of VLPs mentioned above, as it is free of viral immunogenic determinants, and thus will not elicit an anti-viral immune response like conventional VLP vaccines. Following investigations on house dust mite (HDM) [85] and cat [86] allergies, eBPs were applied for the treatment of peanut allergy by displaying Ara h 2 on eBPs’ surface via genetic fusion [65]. Overall, all the VLPs were designed to display either full allergens or their epitopes on the VLP surface. VLP vaccines were generally hypoallergenic and could provide therapeutic and prophylactic effects for food allergies by reducing or preventing the increase of allergen-specific IgE. The VLPs were immunogenic enough to induce strong allergen-specific IgG that they did not require additional adjuvants, except for the study of AAV-VLPs which included alum or Montanide® ISA 51 in the vaccine formulation [60]. Studies on Ara h 2-eBPs also supported the ability of VLPs to activate DCs and induce Th1 response [66].

Here, we elaborate further on the treatment of peanut allergy using CuMVtt-VLPs by Bachmann et al. The vaccine was first designed by chemically coupling extracts of roasted peanut, single allergens Ara h 1 or Ara h 2 to CuMVtt [63]. CuMVtt-VLPs displaying Ara h 2, the major peanut allergen, were shortlisted as a vaccine candidate, but the vaccine design was modified to conjugate Ara h 2 on the VLP surface via genetic fusion [64]. The vaccine candidate was also optimized into mosaic VLPs, in which it was manufactured through the spontaneous assembly of two proteins: CuMVtt and CuMVtt displaying Ara h 2. In vivo subcutaneous immunization on BALB/c mice as a prime-boost regimen demonstrated the prophylactic and therapeutic potentials of the VLP vaccine by protecting the mice against challenge-dependent hypothermia with low anti-Ara h 2-IgE. The therapeutic effect of the vaccine was mechanistically proven through the significant competitive binding of induced anti-Ara h 2 IgG with anti-Ara h 2 IgE for the Ara h 2 epitope. Furthermore, sIgG was shown to be the primary contributor to vaccine efficacy, in which passive transfer of induced IgG-protected peanut-sensitized mice against anaphylaxis. All sIgG subclasses were significantly elevated, with sIgG1 being the dominant response followed by sIgG2a 2 weeks after the booster dose. 30 µg was optimized as the minimum effective dosage, and the study was successfully translated into the first VLP-based clinical trial for FA AIT (Table 3). Phase 1 trial named PROTECT (NCT05476497) commenced in October 2022 to investigate the safety and tolerability of the vaccine in healthy and peanut-allergic patients.

### Lysosomal-Associated Membrane Proteins (LAMPs)

LAMPs are lysosomal integral membrane proteins that have been explored as carriers to facilitate lysosomal and/or endosomal trafficking of antigens, thereby enhancing immunogenicity and reducing the risk of anaphylaxis of allergy vaccines [87]. LAMPs were included as part of the DNA vaccine constructs in AIT of shrimp [28] and peanut [29] allergies. Intradermal immunization of Lit-LAMP-DNA vaccine in shrimp-allergic mice suppressed mast cell activation and allergic symptoms with concomitant induction of robust IgG2a and Th1 responses [28]. Another study showed similar therapeutic efficacy of Ara h1, 2, 3-LAMP DNA vaccine in a peanut-allergic mice model [29]. Two phase 1 trials on peanut-allergic patients (NCT02851277 and NCT03755713) have been conducted (Table 3). Despite a high tolerability of the vaccine and increased allergen-specific IgG and/or IgG4 levels in both trials, there were, unfortunately, no improvements in clinical outcomes [39]. Nevertheless, such technology was also explored in AIT for Japanese red cedar pollen allergy [88], and subsequent phase 1 clinical trial (NCT01966224) indicated that the CryJ2-LAMP DNA vaccine was safe, well-tolerated, with clinical improvement after immunization [89].

### Protamine-Based NPs

Protamine is a naturally occurring polycationic nuclear protein initially derived from the sperm of salmon [90]. With its innate ability to bind and protect DNA during spermatogenesis, protamine self-assembles with negatively charged oligonucleotides, forming nanoparticles called Proticles. Protamine was mainly used as an adjuvant for mRNA vaccines for rabies, metastatic melanoma, and non-small cell lung cancer [91]. In FA, there is one study that utilized Proticles formed through the complex of protamine and CpG-ODN, integrated with Ara h 2 allergen for the treatment of peanut allergy [67]. Immunization of Proticles-Ara h 2 on naïve mice showed Proticles-Ara h 2 was able to prevent allergen sensitization. Proticles-Ara h 2 treated mice had undetectable allergen-specific IgE, a low IL-5/IFN-γ ratio, and increased allergen-specific IgG2a and IgG1 levels. A Th1-driven response was supported by a prior study of protamine on bee venom allergy [92].

## The “Lubricant”—Immunomodulator

Adjuvants that do not function as carriers for the antigens are included in the vaccine formulation as immunomodulators analogous to a “lubricant” that enhances or dampens the immune response. Most of the non-carrier adjuvants used in FA AIT are immunostimulants, such as toll-like receptor ligands and emulsions. Besides these two immunostimulants, there has also been one reported use of a tolerogenic adjuvant, which is the tolerogenic bacterial particles (Table 2).

### Toll-Like Receptor Ligands

Toll-like receptors (TLRs) expressed by innate immune cells are highly specific in recognizing conserved structures associated with foreign microorganisms, also known as pathogen-associated molecular patterns (PAMPs) [44]. PAMPs as ligands of TLRs induce APC activation and promote a strong Th1 response that could counteract the elevated Th2 response in FA [93]. Therefore, TLR ligands such as flagellin and CpG-ODN have been extensively explored as immunogenic pro-Th1 adjuvants for FA AIT.

Flagellin is a major motility protein of bacterial flagella that acts as a TLR-5 agonist, inducing a strong Th1-skewed response. Although flagellin adjuvant is yet to be approved for human clinical use [94], clinical trials of influenza vaccines have reported flagellin to be safe and well-tolerated for incorporation as an adjuvant [95, 96]. AITs for FA have explored the use of flagellin as an immunostimulatory adjuvant. For instance, Schülke et al. showed that type A flagellin genetically fused to allergens like ovalbumin (rfla:OVA) induced stronger DC activation characterized by higher IL-6 and IL-10 production upon stimulation than an equimolar mixture of flagellin and allergen [68]. Subsequently, the administration of the fusion protein reduced allergic symptoms of ovalbumin-sensitized mice, with suppressed sIgE and elevated sIgG2a [69]. Another recent study investigated the role of flagellin in promoting regulatory B cell (Breg) survival by reducing oxidative stress of the cells, contributing to the superior efficacy of including flagellin as the adjuvant in AIT for egg allergy [70].

Cytosine-phosphate-guanine (CpG) motifs found in mammalian DNA are mostly methylated, while the unmethylated CpG motifs commonly present in bacterial DNA act as a TLR-9 agonist that induces a strong Th1-biased immune response [44]. Therefore, synthetic single-stranded oligodeoxynucleotides (ODNs) with unmethylated CpG motifs like CpG-ODNs have been extensively manufactured and evaluated as an adjuvant in vaccine formulation [97]. The phosphate group in CpG-ODN is commonly modified to phosphorothioate to increase stability and resistance to nuclease digestion [98]. Despite being tested in phase I/II clinical trials for infectious diseases and cancers [99], CpG-based drugs are yet to be approved for clinical use [100]. Nevertheless, CpG-ODNs are one of the adjuvants included with other adjuvants in many novel AIT studies for FA including shrimp [25], milk [52], peach [22–24], and peanut [49, 67, 71] allergies. To illustrate the use of CpG-ODNs with other adjuvants, Sampson et al. demonstrated that the co-presentation of CpG-ODNs and allergens through PLGA NPs was crucial in inducing a Th1-skewed response in a peanut allergy mouse model [49]. This finding was further supported by Willemsen et al. that the co-encapsulation of CpG-ODNs and allergen in PLGA NPs displayed superior prophylactic and therapeutic effects compared to separate encapsulation of CpG-ODNs and allergen in NPs [52]. CpG-ODNs were also utilized as a precursor for the formation of Proticles NPs in an AIT for peanut allergy as described earlier [67]. Moreover, the recent study by Wu et al. reported that packaged CpG-ODNs with another adjuvant 1,25-dihydroxyvitamin D3 (VD3) and powdered epicutaneous administration of peanut allergen in a powder-laden dissolvable microneedle array (PLD-MNA) in AIT resulted in reduced sIgE and increased tolerogenic response when compared to conventional intradermal immunotherapy [71].

### Emulsions

Emulsions are a combination of two or more inherently immiscible liquids manufactured through homogenization or microfluidization, resulting in the suspension of one liquid as fine droplets in another liquid [101]. Two-phase emulsions can be categorized into oil-in-water (O/W) and water-in-oil (W/O) emulsions. Other than having an aqueous or water phase and a hydrophobic or oil phase in the formulation, a surfactant is another vital component included to reduce the surface tension between two liquid phases and prevent phase separation over time, thus acting as a stabilizer for the emulsions. Even though emulsions can also serve as carriers for the antigens when encapsulated, this review categorizes emulsions as immunostimulants as the current studies in FA AIT make use of emulsions as immunostimulants rather than as carriers. Emulsions could exert a depot effect on the vaccine antigens and enhance antigen uptake and activation of APCs leading to stronger T and B cell activation [102]. Besides inducing higher antibody titers than unadjuvanted vaccines, emulsions were also found to expand the antibody repertoire that confers cross-protective immunity against non-vaccinated antigenic variants, based on evidence from clinical studies on influenza vaccines [103].

The common W/O emulsion used in research is Freund’s adjuvant, which consists of paraffin oil and lanolin as the oil phase components [102]. However, Freund’s adjuvant is no longer approved for human use despite its superior ability to induce antibody titer and immune persistence due to serious adverse reactions like ulcers at the injection sites and granuloma formation when incorporated in the influenza vaccine [104]. Therefore, except for the AAV-VLP AIT which investigated a type of Freund’s adjuvant, Montanide® ISA 51 [60], all other FA AIT studies used the safer O/W emulsion.

The novel adjuvants of O/W emulsions currently approved for human clinical use are MF59 and AS03 [101]. Both are squalene-based emulsions included in licensed influenza vaccines with substantial clinically proven effectiveness and a well-established safety profile [105, 106]. The mimetic of MF59, AddaVax, which has been known to induce a balanced Th1/Th2 immunity, was investigated as the adjuvant for the treatment of allergic contact dermatitis [107]. In FA AIT, Baker et al. constructed an O/W emulsion with ultrapure soybean oil as the oil phase and ethanol as the water phase [72]. Cetylpyridinium chloride and Tween 80 (Polysorbate 80) were included in the preparation as surfactants. Intranasal immunization of the resulting W/O emulsion mixed with peanut extract protected peanut-sensitized mice from developing oral and systemic allergic reactions [72, 73]. Th1, Th17, and Treg responses were increased with a concomitant decrease in Th2 response. There was also a marked decrease in peanut-specific IgE and IgG1 but a significant increase in peanut-specific IgG2a and IgG2b in mice treated with W/O emulsion and peanut extract.

In another study using the same emulsion for the treatment of milk allergy, four monthly intranasal administrations of casein with emulsion conferred sustained unresponsiveness for at least 16 weeks after the last immunization [74]. Despite cow’s milk-specific IgE in the treatment group not being significantly different from the PBS group after challenge, the therapeutic effect on mice can be partly accounted for by the 20-fold increase in the sIgG2a that could inhibit sIgE. Interestingly, the same emulsion approach also showed potential therapeutic efficacy in a multi-FA setting, in which co-administration of emulsion with peanut and ovalbumin protected mice with both peanut and egg allergies from developing anaphylaxis [75]. This study also demonstrated the cross-protective ability of a single allergen using peanut extract with emulsion to confer protection against peanut and egg allergy, indicating a bystander effect with sustained unresponsiveness for at least eight weeks.

### Tolerogenic Bacterial Particles (TBP)

Tolerogenic adjuvants have been recently investigated by Sørensen et al. in AIT for egg [76] and tree nut [77] allergies. Tolerogenic bacterial particles (TBP) derived from a probiotic *Lactococcus lactis* were utilized as the tolerogenic adjuvant and mixed with targeted allergens as a form of sublingual immunotherapy. In egg AIT, the sublingual administration of the TBP and ovalbumin mixture loaded on nanofiber-based mucoadhesive patches on ovalbumin-sensitized piglets demonstrated a synergistic effect of TBP and the allergen in reducing allergic symptoms and increasing Treg response [76]. With the main goal of the TBP adjuvant to induce Treg responses, stimulation of PBMCs from tree-nut-allergic patients with TBP mixed with walnut extract showed a significant increase in the IL-10/IL-4 and IFN-γ/IL-4 ratio, indicating that TBP was not only able to suppress Th2 and induce Treg but also enhanced Th1 responses [77]. Overall, both studies highlighted the potential of TBP as a novel tolerogenic adjuvant with a slight Th1-biased response when co-administered with food allergens.

## Summary and Outlook

The advancement of next-generation AIT for FA has been significant over the past decade. To describe the overall approach of next-generation AIT, a “cargo-truck-lubricant” analogy is proposed in which the “cargo” refers to the allergen, the “truck” refers to the carrier, and the “lubricant” refers to the immunomodulators (Fig. 2). Novel AIT strategies targeting the “cargo” administer the food allergens in the form of protein or nucleic acids. The main goal of various strategies is to reduce the allergenicity of the allergen, either through genetic or chemical modification of the full-length allergen, or the administration of single or multiple epitopes as a polypeptide-based AIT. On the other hand, next-generation AIT strategies targeting the “truck” and/or “lubricant” explore a variety of adjuvants to promote high immunogenicity of the vaccine, as well as the effect of different combinations of adjuvants to further enhance the adjuvant effects. Nevertheless, potential side effects of incorporating additional adjuvants in the vaccine formulation should not be disregarded. Among the big nine food allergens, peanuts, tree nuts, milk, egg, wheat, soy, fish, shellfish, and sesame [108], next-generation AITs were extensively investigated for peanut allergy. For instance, animal studies of AIT based on virus-like particles, PLGA NPs, and LAMP have been successfully translated to clinical trials for peanut allergy. While there were also several pre-clinical studies on egg, milk, shellfish, fish, and tree nut allergies, next-generation AIT is yet to be explored for soy, wheat, and sesame allergies. The hindered progress in clinical translation can be attributed to several key hurdles. First, the heterogeneity in trial designs for each AIT product, including differences in AIT formulation, administration route, and treatment schedule, has restricted the generalizability across different FA and posed challenges from an economic standpoint [109]. Second, pharmaceutical companies may be reluctant to develop novel AIT products due to small market size or stringent regulations, particularly for FA with low prevalence. This may be due to the difficulty in attaining sufficient sample size for the validation of AIT efficacy through randomized, placebo-controlled food challenge to comply with good clinical practice (GCP), which is often a mandatory requirement in countries like the EU member states [110]. Third, while the inclusion and exclusion criteria in clinical trials are essential to minimize bias, the highly selected patient population often does not represent the real-world conditions, as potential confounding factors like comorbidities and smoking may impact AIT outcomes [111].

AIT represents the most promising frontiers in food allergy management. However, the potential of the proposed innovative vaccine formulations for FA AIT to achieve sustained unresponsiveness remains largely unanswered due to limited long-term follow-up studies, both in animal models and clinical trials. A few studies have explored and shown promising results of sustained unresponsiveness in milk, peanut, and multi-FA, but the duration among the studies is inconsistent [46, 74, 75]. On the other hand, the safety profile of these next-generation AIT is still lacking. Nevertheless, the safety of similar formulations, for example, mRNA-LNP vaccine, has been investigated in other diseases like COVID-19 that show their generally high safety profiles with very low incidence of serious adverse events [112]. Rare serious outcomes such as myocardial infarction, stroke, myocarditis, and anaphylaxis were reported but without significant association to the vaccination. Another study has found a positive association between the risk of anaphylaxis and the immunization of an mRNA-LNP vaccine targeting a recent mutant of COVID-19 [113]. It is also important to consider the risk and benefit of including additional adjuvants based on their respective clinical data in the past, such as a reported case of PEG allergy in a patient immunized with an mRNA-LNP vaccine containing PEG as an LNP stabilizer [114].

Therefore, further research effort directed at translating pre-clinical findings into clinical trials to unravel the long-term safety and efficacy of novel AIT vaccines in allergic patients is much warranted. Collaborative studies will help to overcome the hurdles on extending these AIT concepts into other FAs beyond peanut allergy. While the majority of the current studies focused on single FAs, multi-FA is common in both children and adults, and there is an unmet need in studying next-generation AIT for multi-FA. Increasing cost-effectiveness associated with next-generation AITs, streamlining treatment delivery, and increasing public awareness of AIT in FAs will greatly help to ensure that these therapies are effective and safe while accessible to all who need them.

## Data Availability

No datasets were generated or analysed during the current study.

## Abbreviations

AAV: Adeno-associated virus
AIT: Allergen-specific immunotherapy
AK: Arginine kinase
APCs: Antigen-presenting cells
BLG: β-Lactoglobulin
Bregs: Regulatory B cells
CpG: Cytosine-phosphate/phosphorothioate-guanine
CuMVtt: Cucumber mosaic virus including tetanus toxin epitopes
DCs: Dendritic cells
eBP: Enveloped bioparticle
FA: Food allergy
FDA: Food and Drug Administration
HDM: House dust mite
HEL: Hen egg lysozyme
LAMPs: Lysosomal-associated membrane proteins
LNPs: Lipid-based nanoparticles
LSECs: Liver sinusoidal endothelial cells
NMR: Nuclear magnetic resonance
NoVLPs: Norovirus-like particles
ODNs: Oligodeoxynucleotides
OIT: Oral immunotherapy
OUtMATCH: Omalizumab as Monotherapy and as Adjunct Therapy to Multi-Allergen Oral Immunotherapy in Food-Allergic Children and Adults
O/W: Oil-in-water
PAMPs: Pathogen-associated molecular patterns
PEG: Polyethylene glycol
PLD-MNA: Power-laden, dissolvable microneedle array
PLGA: Poly(lactic-co-glycolic) acid
PVMA: Poly(methyl vinyl ether-co-maleic anhydride)
SAEs: Serious adverse events
SARS-CoV-2: Severe acute respiratory syndrome coronavirus 2
sIgE: Antigen-specific IgE
Siglecs: Sialic acid-binding immunoglobulin-type lectins
STALs: Siglec-engaging tolerance-inducing antigenic liposomes
SU: Sustained unresponsiveness
TBPs: Tolerogenic bacterial particles
Th2: Type 2 T helper
TLRs: Toll-like receptors
TM: Tropomyosin
Tregs: Regulatory T cells
TuMV: Turnip mosaic virus
VD3: 1,25-Dihydroxyvitamin D3
VLPs: Virus-like particles
W/O: Water-in-oil

## References

[1] Gupta RS, Springston EE, Warrier MR, Smith B, Kumar R, Pongracic J et al (2011) The prevalence, severity, and distribution of childhood food allergy in the United States. Pediatrics 128(1):e9-17 21690110 10.1542/peds.2011-0204

[2] Warren CM, Jiang J, Gupta RS (2020) Epidemiology and burden of food allergy. Curr Allergy Asthma Rep 20(2):6 32067114 10.1007/s11882-020-0898-7 PMC7883751

[3] Osborne NJ, Koplin JJ, Martin PE, Gurrin LC, Lowe AJ, Matheson MC et al (2011) Prevalence of challenge-proven IgE-mediated food allergy using population-based sampling and predetermined challenge criteria in infants. Journal of Allergy and Clinical Immunology 127(3):668-76.e1–2 21377036 10.1016/j.jaci.2011.01.039

[4] Hu Y, Chen J, Li H (2010) Comparison of food allergy prevalence among Chinese infants in Chongqing, 2009 versus 1999. Pediatr Int 52(5):820–824 20487367 10.1111/j.1442-200X.2010.03166.x

[5] Peters RL, Koplin JJ, Gurrin LC, Dharmage SC, Wake M, Ponsonby AL et al (2017) The prevalence of food allergy and other allergic diseases in early childhood in a population-based study: HealthNuts age 4-year follow-up. J Allergy Clin Immunol 140(1):145–53.e8 10.1016/j.jaci.2017.02.019 28514997

[6] Schoemaker AA, Sprikkelman AB, Grimshaw KE, Roberts G, Grabenhenrich L, Rosenfeld L et al (2015) Incidence and natural history of challenge-proven cow’s milk allergy in European children—EuroPrevall birth cohort. Allergy 70(8):963–972 25864712 10.1111/all.12630

[7] Tsabouri S, Triga M, Makris M, Kalogeromitros D, Church MK, Priftis KN (2012) Fish and shellfish allergy in children: review of a persistent food allergy. Pediatr Allergy Immunol 23(7):608–615 22554093 10.1111/j.1399-3038.2012.01275.x

[8] Holgate S, Casale T, Wenzel S, Bousquet J, Deniz Y, Reisner C (2005) The anti-inflammatory effects of omalizumab confirm the central role of IgE in allergic inflammation. J Allergy Clin Immunol 115(3):459–465 15753888 10.1016/j.jaci.2004.11.053

[9] Kulis MD, Humphrey JR, Krempski JW, Kim EH, Smeekens JM (2025) Anti-IgE therapy versus allergen-specific immunotherapy for food allergy: weighing the pros and cons. Front Immunol 16:1617153 40766311 10.3389/fimmu.2025.1617153 PMC12321543

[10] Wood RA, Togias A, Sicherer SH, Shreffler WG, Kim EH, Jones SM et al (2024) Omalizumab for the treatment of multiple food allergies. N Engl J Med 390(10):889–899 38407394 10.1056/NEJMoa2312382 PMC11193494

[11] Burks AW, Jones SM, Wood RA, Fleischer DM, Sicherer SH, Lindblad RW et al (2012) Oral immunotherapy for treatment of egg allergy in children. N Engl J Med 367(3):233–243 22808958 10.1056/NEJMoa1200435 PMC3424505

[12] Nurmatov U, Dhami S, Arasi S, Pajno GB, Fernandez-Rivas M, Muraro A et al (2017) Allergen immunotherapy for IgE-mediated food allergy: a systematic review and meta-analysis. Allergy 72(8):1133–1147 28058751 10.1111/all.13124

[13] Romantsik O, Tosca MA, Zappettini S, Calevo MG (2018) Oral and sublingual immunotherapy for egg allergy. Cochrane Database Syst Rev 4(4):CD010638 29676439 10.1002/14651858.CD010638.pub3 PMC6494514

[14] Wai CY, Leung NY, Ho MH, Gershwin LJ, Shu SA, Leung PS et al (2014) Immunization with hypoallergens of shrimp allergen tropomyosin inhibits shrimp tropomyosin-specific IgE reactivity. PLoS ONE 9(11):e111649 25365343 10.1371/journal.pone.0111649 PMC4218792

[15] Swoboda I, Bugajska-Schretter A, Linhart B, Verdino P, Keller W, Schulmeister U et al (2007) A recombinant hypoallergenic parvalbumin mutant for immunotherapy of IgE-mediated fish allergy. J Immunol 178(10):6290–6296 17475857 10.4049/jimmunol.178.10.6290

[16] Zuidmeer-Jongejan L, Huber H, Swoboda I, Rigby N, Versteeg SA, Jensen BM et al (2015) Development of a hypoallergenic recombinant parvalbumin for first-in-man subcutaneous immunotherapy of fish allergy. Int Arch Allergy Immunol 166(1):41–51 25765512 10.1159/000371657

[17] Zhang Z, Xiao H, Zhou P (2019) Allergenicity suppression of tropomyosin from *Exopalaemon modestus* by glycation with saccharides of different molecular sizes. Food Chem 288:268–275 30902292 10.1016/j.foodchem.2019.03.019

[18] Zhang Z, Li X-M, Li Z, Lin H (2021) Investigation of glycated shrimp tropomyosin as a hypoallergen for potential immunotherapy. Food Funct 12(6):2750–2759 33683237 10.1039/d0fo03039b

[19] Anzengruber J, Bublin M, Bonisch E, Janesch B, Tscheppe A, Braun ML et al (2017) *Lactobacillus buchneri* S-layer as carrier for an *Ara h 2*-derived peptide for peanut allergen-specific immunotherapy. Mol Immunol 85:81–88 28212503 10.1016/j.molimm.2017.02.005 PMC5386144

[20] Wai CY, Leung NY, Leung PS, Chu KH (2016) T cell epitope immunotherapy ameliorates allergic responses in a murine model of shrimp allergy. Clin Exp Allergy 46(3):491–503 26610061 10.1111/cea.12684

[21] Candreva AM, Smaldini PL, Cauerhff A, Petruccelli S, Docena GH (2021) A novel approach to ameliorate experimental milk allergy based on the oral administration of a short soy cross-reactive peptide. Food Chem 346:128926 33484948 10.1016/j.foodchem.2020.128926

[22] Rodriguez MJ, Ramos-Soriano J, Perkins JR, Mascaraque A, Torres MJ, Gomez F et al (2019) Glycosylated nanostructures in sublingual immunotherapy induce long-lasting tolerance in LTP allergy mouse model. Sci Rep 9(1):4043 30858392 10.1038/s41598-019-40114-7 PMC6411722

[23] Nunez R, Rodriguez MJ, Lebron-Martin C, Martin-Astorga MDC, Palomares F, Ramos-Soriano J et al (2022) Methylation changes induced by glycodendropeptide immunotherapy and associated with tolerance in mice. Front Immunol 13:1094172 36643916 10.3389/fimmu.2022.1094172 PMC9832389

[24] Nunez R, Rodriguez MJ, Lebron-Martin C, Martin-Astorga MDC, Ramos-Soriano J, Rojo J et al (2023) A synthetic glycodendropeptide induces methylation changes on regulatory T cells linked to tolerant responses in anaphylactic mice. Front Immunol 14:1165852 37334360 10.3389/fimmu.2023.1165852 PMC10272618

[25] Hong J, Gao Q, Xiao X, Cao H, Yuan R, Liu Z et al (2020) T cell epitope of arginine kinase with CpG co-encapsulated nanoparticles attenuates shrimp allergen-induced Th2-bias food allergy. Biosci Biotechnol Biochem 84(4):804–814 31795812 10.1080/09168451.2019.1699395

[26] Smeekens JM, Kesselring JR, Frizzell H, Bagley KC, Kulis MD (2022) Induction of food-specific IgG by gene gun-delivered DNA vaccines. Front Allergy 3:969337 36340020 10.3389/falgy.2022.969337 PMC9632862

[27] Wai CYY, Leung NYH, Leung PSC, Chu KH (2019) Modulating shrimp tropomyosin-mediated allergy: hypoallergen DNA vaccines induce regulatory T cells to reduce hypersensitivity in mouse model. Int J Mol Sci. 10.3390/ijms20184656 31546958 10.3390/ijms20184656 PMC6769673

[28] Kubo K, Takeda S, Uchida M, Maeda M, Endo N, Sugahara S et al (2022) Lit-LAMP-DNA vaccine for shrimp allergy prevents anaphylactic symptoms in a murine model. Int Immunopharmacol 113(Pt A):109394 36334369 10.1016/j.intimp.2022.109394

[29] Li X-M, Song Y, Su Y, Heiland T, Sampson HA (2015) Immunization with ARA h1,2,3-lamp-vax peanut vaccine blocked IgE mediated-anaphylaxis in a peanut allergic murine model. J Allergy Clin Immunol 135(2):AB167

[30] Xu X, Wang X, Liao YP, Luo L, Xia T, Nel AE (2023) Use of a liver-targeting immune-tolerogenic mRNA lipid nanoparticle platform to treat peanut-induced anaphylaxis by single- and multiple-epitope nucleotide sequence delivery. ACS Nano 17(5):4942–4957 36853930 10.1021/acsnano.2c12420 PMC10019335

[31] Larsen JE, Lund O, Nielsen M (2006) Improved method for predicting linear B-cell epitopes. Immunome Res 2(1):2 10.1186/1745-7580-2-2 PMC1479323 16635264

[32] Emini EA, Hughes JV, Perlow DS, Boger J (1985) Induction of hepatitis A virus-neutralizing antibody by a virus-specific synthetic peptide. J Virol 55(3):836–839 2991600 10.1128/jvi.55.3.836-839.1985 PMC255070

[33] Kolaskar AS, Tongaonkar PC (1990) A semi-empirical method for prediction of antigenic determinants on protein antigens. FEBS Lett 276(1–2):172–174 1702393 10.1016/0014-5793(90)80535-q

[34] Sharp MF, Taki AC, Ruethers T, Stephen JN, Daly NL, Lopata AL et al (2021) IgE and IgG(4) epitopes revealed on the major fish allergen *Lat c 1*. Mol Immunol 131:155–163 33423763 10.1016/j.molimm.2020.12.033

[35] Suprun M, Sicherer SH, Wood RA, Jones SM, Leung DYM, Burks AW et al (2022) Mapping sequential IgE-binding epitopes on major and minor egg allergens. Int Arch Allergy Immunol 183(3):249–261 34818647 10.1159/000519618

[36] Fu L, Wang C, Wang J, Ni S, Wang Y (2019) Maillard reaction with ribose, galacto-oligosaccharide, or chitosan-oligosaccharide reduces allergenicity of shrimp tropomyosin by inducing conformational changes. Food Chem 274:789–795 30373009 10.1016/j.foodchem.2018.09.068

[37] Ribeiro-Viana R, Garcia-Vallejo JJ, Collado D, Perez-Inestrosa E, Bloem K, van Kooyk Y et al (2012) BODIPY-labeled DC-SIGN-targeting glycodendrons efficiently internalize and route to lysosomes in human dendritic cells. Biomacromol 13(10):3209–3219 10.1021/bm300998c 22920925

[38] Renand A, Newbrough S, Wambre E, DeLong JH, Robinson D, Kwok WW (2024) Arginine kinase *Pen m 2* as an important shrimp allergen recognized by th2 cells. J Allergy Clin Immunol 134(6):1456–9.e7 10.1016/j.jaci.2014.07.048 PMC4349443 25224098

[39] Ferslew BC, Smulders R, Zhu T, Blauwet MB, Kusawake T, Spence A et al (2024) Safety and Immunopharmacology of ASP0892 in adults or adolescents with peanut allergy: two randomized trials allergy. Allergy 79(2):456–70 10.1111/all.15931 38010254

[40] Polack FP, Thomas SJ, Kitchin N, Absalon J, Gurtman A, Lockhart S et al (2020) Safety and efficacy of the BNT162b2 mRNA COVID-19 vaccine. N Engl J Med 383(27):2603–2615 33301246 10.1056/NEJMoa2034577 PMC7745181

[41] Krienke C, Kolb L, Diken E, Streuber M, Kirchhoff S, Bukur T et al (2021) A noninflammatory mrna vaccine for treatment of experimental autoimmune encephalomyelitis. Science 371(6525):145–153 33414215 10.1126/science.aay3638

[42] Petrovsky N, Aguilar JC (2004) Vaccine adjuvants: current state and future trends. Immunol Cell Biol 82(5):488–496 15479434 10.1111/j.0818-9641.2004.01272.x

[43] Rad LM, Arellano G, Podojil JR, O’Konek JJ, Shea LD, Miller SD (2024) Engineering nanoparticle therapeutics for food allergy. J Allergy Clin Immunol 153(3):549–559 37926124 10.1016/j.jaci.2023.10.013 PMC10939913

[44] Lin Y-J, Zimmermann J, Schülke S (2024) Novel adjuvants in allergen-specific immunotherapy: where do we stand? Front Immunol. 10.3389/fimmu.2024.1348305 38464539 10.3389/fimmu.2024.1348305 PMC10920236

[45] Orgel KA, Duan S, Wright BL, Maleki SJ, Wolf JC, Vickery BP et al (2017) Exploiting CD22 on antigen-specific b cells to prevent allergy to the major peanut allergen Ara h 2. Allergy Clin Immunol 139(1):366–9 e2 10.1016/j.jaci.2016.06.053 PMC5222808 27554819

[46] Hardy LC, Smeekens JM, Raghuwanshi D, Sarkar S, Daskhan GC, Rogers S et al (2022) Targeting CD22 on memory b cells to induce tolerance to peanut allergens. J Allergy Clin Immunol 150(6):1476-1485.e4 35839842 10.1016/j.jaci.2022.06.022 PMC9813968

[47] Duan S, Arlian BM, Nycholat CM, Wei Y, Tateno H, Smith SA et al (2021) Nanoparticles displaying allergen and siglec-8 ligands suppress IgE-FcepsilonRI-mediated anaphylaxis and desensitize mast cells to subsequent antigen challenge. J Immunol Res 206(10):2290–2300 10.4049/jimmunol.1901212 PMC8113104 33911007

[48] Kiwamoto T, Kawasaki N, Paulson JC, Bochner BS (2012) Siglec-8 as a druggable target to treat eosinophil and mast cell-associated conditions. Pharmacol Ther 135(3):327–336 22749793 10.1016/j.pharmthera.2012.06.005 PMC3587973

[49] Srivastava KD, Siefert A, Fahmy TM, Caplan MJ, Li XM, Sampson HA (2016) Investigation of peanut oral immunotherapy with CpG/peanut nanoparticles in a murine model of peanut allergy. J Allergy Clin Immunol. 2016;138(2):536–43 e4. 10.1016/j.jaci.2016.01.047 27130858

[50] Kostadinova AI, Middelburg J, Ciulla M, Garssen J, Hennink WE, Knippels LMJ et al (2018) PLGA nanoparticles loaded with beta-lactoglobulin-derived peptides modulate mucosal immunity and may facilitate cow’s milk allergy prevention. Eur J Pharmacol 818:211–220 29079360 10.1016/j.ejphar.2017.10.051

[51] Liu M, Thijssen S, van Nostrum CF, Hennink WE, Garssen J, Willemsen LEM (2022) Inhibition of cow’s milk allergy development in mice by oral delivery of beta-lactoglobulin-derived peptides loaded in PLGA nanoparticles is associated with systemic whey-specific immune silencing. Clin Exp Allergy 52(1):137–148 34145667 10.1111/cea.13967 PMC9291823

[52] Liu M, Thijssen S, Hennink WE, Garssen J, van Nostrum CF, Willemsen LEM (2022) Oral pretreatment with beta-lactoglobulin derived peptide and CpG co-encapsulated in PLGA nanoparticles prior to sensitizations attenuates cow’s milk allergy development in mice. Front Immunol 13:1053107 36703973 10.3389/fimmu.2022.1053107 PMC9872660

[53] Hughes KR, Saunders MN, Landers JJ, Janczak KW, Turkistani H, Rad LM et al (2022) Masked delivery of allergen in nanoparticles safely attenuates anaphylactic response in murine models of peanut allergy. Front Allergy 3:829605 35386645 10.3389/falgy.2022.829605 PMC8974743

[54] Saunders MN, Rad LM, Williams LA, Landers JJ, Urie RR, Hocevar SE, Quiros M, Chiang MY, Angadi AR, Janczak KW, Bealer EJ (2024) Allergen-encapsulating nanoparticles reprogram pathogenic allergen-specific Th2 cells to suppress food allergy. Adv Healthc Mater 14(5):2400237 10.1002/adhm.202400237 PMC11527797 38691819

[55] Saunders MN, Rival CM, Mandal M, Cramton K, Rad LM, Janczak KW et al (2024) Immunotherapy with biodegradable nanoparticles encapsulating the oligosaccharide galactose-alpha-1,3-galactose enhances immune tolerance against alpha-gal sensitization in a murine model of alpha-gal syndrome. Front Allergy 5:1437523 39183976 10.3389/falgy.2024.1437523 PMC11341473

[56] Sakurai R, Iwata H, Gotoh M, Ogino H, Takeuchi I, Makino K et al (2024) Application of PLGA-PEG-PLGA nanoparticles to percutaneous immunotherapy for food allergy. Molecules. 10.3390/molecules29174123 39274971 10.3390/molecules29174123 PMC11397245

[57] De SRJ, Irache JM, Camacho AI, Gastaminza G, Sanz ML, Ferrer M et al (2014) Immunogenicity of peanut proteins containing poly(anhydride) nanoparticles. Clin Vaccine Immunol 21(8):1106–1112 24899075 10.1128/CVI.00359-14 PMC4135921

[58] Gamazo C, Garcia-Azpiroz M, Souza Reboucas J, Gastaminza G, Ferrer M, Irache JM (2017) Oral immunotherapy using polymeric nanoparticles loaded with peanut proteins in a murine model of fatal anaphylaxis. Immunotherapy 9(15):1205–1217 29130802 10.2217/imt-2017-0111

[59] Pereira MA, Reboucas JS, Ferraz-Carvalho RS, de Redin IL, Guerra PV, Gamazo C et al (2018) Poly(anhydride) nanoparticles containing cashew nut proteins can induce a strong Th1 and Treg immune response after oral administration. Eur J Pharm Biopharm 127:51–60 29428795 10.1016/j.ejpb.2018.02.011

[60] Manzano-Szalai K, Thell K, Willensdorfer A, Weghofer M, Pfanzagl B, Singer J et al (2014) Adeno-associated virus-like particles as new carriers for B-cell vaccines: testing immunogenicity and safety in BALB/c mice. Viral Immunol 27(9):438–448 25247267 10.1089/vim.2014.0059 PMC4217042

[61] Aguilar Conde A (2020) Inmunoterapia Basada en NoVLPs como Tratamiento Potencial para la Alergia al Melocotón. Trabajo Fin de Grado / Proyecto Fin de Carrera, E.T.S. de Ingeniería Agronómica, Alimentaria y de Biosistemas (UPM), Madrid

[62] Pazos-Castro D, Margain C, Gonzalez-Klein Z, Amores-Borge M, Yuste-Calvo C, Garrido-Arandia M et al (2022) Suitability of potyviral recombinant virus-like particles bearing a complete food allergen for immunotherapy vaccines. Front Immunol 13:986823 36159839 10.3389/fimmu.2022.986823 PMC9492988

[63] Storni F, Zeltins A, Balke I, Heath MD, Kramer MF, Skinner MA et al (2020) Vaccine against peanut allergy based on engineered virus-like particles displaying single major peanut allergens. J Allergy Clin Immunol 145(4):1240–53 e3 10.1016/j.jaci.2019.12.007 31866435

[64] Sobczak JM, Krenger PS, Storni F, Mohsen MO, Balke I, Resevica G et al (2023) The next generation virus-like particle platform for the treatment of peanut allergy. Allergy 78(7):1980–1996 36883475 10.1111/all.15704

[65] Castenmiller C, Stigler M, Kirpas ME, Versteeg S, Akkerdaas JH, Pena-Castellanos G et al (2023) Plant-based enveloped *Ara h 2* bioparticles display exceptional hypo-allergenicity. Clin Exp Allergy 53(5):577–581 36779573 10.1111/cea.14294 PMC10402690

[66] Castenmiller C, Nagy NA, Kroon PZ, Auger L, Desgagnes R, Martel C et al (2023) A novel peanut allergy immunotherapy: plant-based enveloped *Ara h 2* bioparticles activate dendritic cells and polarize T cell responses to Th1. World Allergy Organ J 16(11):100839 38020282 10.1016/j.waojou.2023.100839 PMC10679945

[67] Pali-Scholl I, Szollosi H, Starkl P, Scheicher B, Stremnitzer C, Hofmeister A et al (2013) Protamine nanoparticles with CpG-oligodeoxynucleotide prevent an allergen-induced Th2-response in BALB/c mice. Eur J Pharm Biopharm 85(3 Pt A):656–664 23523543 10.1016/j.ejpb.2013.03.003

[68] Schulke S, Waibler Z, Mende MS, Zoccatelli G, Vieths S, Toda M et al (2010) Fusion protein of TLR5-ligand and allergen potentiates activation and IL-10 secretion in murine myeloid dendritic cells. Mol Immunol 48(1–3):341–350 20965571 10.1016/j.molimm.2010.07.006

[69] Schulke S, Burggraf M, Waibler Z, Wangorsch A, Wolfheimer S, Kalinke U et al (2011) A fusion protein of flagellin and ovalbumin suppresses the TH2 response and prevents murine intestinal allergy. J Allergy Clin Immunol 128(6):1340-1348.e12 21872305 10.1016/j.jaci.2011.07.036

[70] Zeng HT, Liu Y, Zhao M, Liu JQ, Jin QR, Liu ZQ et al (2022) Modulating oxidative stress in B cells promotes immunotherapy in food allergy. Oxid Med Cell Longev 2022:3605977 35096267 10.1155/2022/3605977 PMC8799367

[71] Yu Y, Kiran Kumar MN, Wu MX (2020) Delivery of allergen powder for safe and effective epicutaneous immunotherapy. J Allergy Clin Immunol 145(2):597–609 31783055 10.1016/j.jaci.2019.11.022 PMC7430154

[72] O’Konek JJ, Landers JJ, Janczak KW, Goel RR, Mondrusov AM, Wong PT et al (2018) Nanoemulsion adjuvant-driven redirection of T(H)2 immunity inhibits allergic reactions in murine models of peanut allergy. J Allergy Clin Immunol 141(6):2121–2131 29655584 10.1016/j.jaci.2018.01.042

[73] O’Konek JJ, Baker JR Jr (2020) Treatment of allergic disease with nanoemulsion adjuvant vaccines. Allergy 75(1):246–249 31298741 10.1111/all.13977 PMC6952560

[74] O’Konek JJ, Landers JJ, Janczak KW, Lindsey HK, Mondrusov AM, Totten TD et al (2020) Intranasal nanoemulsion vaccine confers long-lasting immunomodulation and sustained unresponsiveness in a murine model of milk allergy. Allergy 75(4):872–881 31557317 10.1111/all.14064

[75] Farazuddin M, Landers JJ, Janczak KW, Lindsey HK, Finkelman FD, Baker JR Jr et al (2021) Mucosal nanoemulsion allergy vaccine suppresses alarmin expression and induces bystander suppression of reactivity to multiple food allergens. Front Immunol 12:599296 33717078 10.3389/fimmu.2021.599296 PMC7946984

[76] Sorensen P, Turanek-Knotigova P, Masek J, Kotoucek J, Hubatka F, Maskova E et al (2021) Short-course sublingual immunotherapy by mucoadhesive patch and tolerogenic particle enhanced allergen presentation. Clin Exp Allergy 51(6):853–857 33682209 10.1111/cea.13862

[77] Palomares F, Mayorga C, Leenhouts K, Perez-Sanchez N, Gomez F, Torres MJ et al (2023) *Lactococcus lactis*-derived microparticles mixed with allergen shift immune responses towards a regulatory profile in tree-nut allergic patients. Allergy 78(3):851–855 36463436 10.1111/all.15607

[78] De Souza Reboucas J, Esparza I, Ferrer M, Sanz ML, Irache JM, Gamazo C (2012) Nanoparticulate adjuvants and delivery systems for allergen immunotherapy. J Biomed Biotechnol 2012:474605 22496608 10.1155/2012/474605 PMC3303624

[79] Pohlit H, Bellinghausen I, Frey H, Saloga J (2017) Recent advances in the use of nanoparticles for allergen-specific immunotherapy. Allergy 72(10):1461–1474 28474379 10.1111/all.13199

[80] Zielinska A, Carreiro F, Oliveira AM, Neves A, Pires B, Venkatesh DN et al (2020) Polymeric nanoparticles: production, characterization, toxicology and ecotoxicology. Molecules 25(16):3731 32824172 10.3390/molecules25163731 PMC7464532

[81] Pechsrichuang P, Namwongnao S, Jacquet A (2021) Bioengineering of virus-like particles for the prevention or treatment of allergic diseases. Allergy Asthma Immunol Res 13(1):23–41 33191675 10.4168/aair.2021.13.1.23 PMC7680827

[82] Anzaghe M, Schulke S, Scheurer S (2018) Virus-like particles as carrier systems to enhance immunomodulation in allergen immunotherapy. Curr Allergy Asthma Rep 18(12):71 30362017 10.1007/s11882-018-0827-1

[83] Gharailoo Z, Plattner K, Augusto G, Engeroff P, Vogel M, Bachmann MF (2024) Generation of a virus-like particles based vaccine against IgE. Allergy 79(8):2207–2221 38445568 10.1111/all.16090

[84] Jensen-Jarolim E, Roth-Walter F, Jordakieva G, Pali-Scholl I (2021) Allergens and adjuvants in allergen immunotherapy for immune activation, tolerance, and resilience. J Allergy Clin Immunol Pract 9(5):1780–1789 33753052 10.1016/j.jaip.2020.12.008

[85] Gomord V, Stordeur V, Fitchette AC, Fixman ED, Tropper G, Garnier L et al (2020) Design, production and immunomodulatory potency of a novel allergen bioparticle. PLoS ONE 15(12):e0242867 33259521 10.1371/journal.pone.0242867 PMC7707610

[86] Busold S, Aglas L, Menage C, Auger L, Desgagnes R, Faye L et al (2022) *Fel d 1* surface expression on plant-made ebioparticles combines potent immune activation and hypoallergenicity. Allergy 77(10):3124–3126 35916123 10.1111/all.15464 PMC10286754

[87] Mennini M, Piccirillo M, Furio S, Valitutti F, Ferretti A, Strisciuglio C et al (2024) Probiotics and other adjuvants in allergen-specific immunotherapy for food allergy: a comprehensive review. Front Allergy 5:1473352 39450374 10.3389/falgy.2024.1473352 PMC11499231

[88] Su Y, Connolly M, Marketon A, Heiland T (2016) CryJ-LAMP DNA vaccines for Japanese red cedar allergy induce robust Th1-type immune responses in murine model. J Immunol Res 2016(1):4857869 27239481 10.1155/2016/4857869 PMC4867073

[89] Su Y, Romeu-Bonilla E, Anagnostou A, Fitz-Patrick D, Hearl W, Heiland T (2017) Safety and long-term immunological effects of CryJ2-LAMP plasmid vaccine in Japanese Red Cedar atopic subjects: a phase I study. Hum Vaccin Immunother 13(12):2804–2813 28605294 10.1080/21645515.2017.1329070 PMC5718801

[90] Scheicher B, Schachner-Nedherer AL, Zimmer A (2015) Protamine-oligonucleotide nanoparticles: recent advances in drug delivery and drug targeting. Eur J Pharm Sci 75:54–59 25896372 10.1016/j.ejps.2015.04.009

[91] Barbier AJ, Jiang AY, Zhang P, Wooster R, Anderson DG (2022) The clinical progress of mRNA vaccines and immunotherapies. Nat Biotechnol 40(6):840–854 35534554 10.1038/s41587-022-01294-2

[92] Martinez Gomez JM, Fischer S, Csaba N, Kundig TM, Merkle HP, Gander B et al (2007) A protective allergy vaccine based on CpG- and protamine-containing PLGA microparticles. Pharm Res 24(10):1927–1935 17541735 10.1007/s11095-007-9318-0

[93] Hennessy EJ, Parker AE, O’Neill LA (2010) Targeting toll-like receptors: emerging therapeutics? Nat Rev Drug Discovery 9(4):293–307 20380038 10.1038/nrd3203

[94] Pang S, Liu M, Wang L, Shao M, Zhu G, Duan Q (2024) Differential adjuvant activity by flagellins from *Escherichia coli*, *Salmonella enterica* serotype typhimurium, and *Pseudomonas aeruginosa*. Vaccines 12(11):1212 39591115 10.3390/vaccines12111212 PMC11598095

[95] Turley CB, Rupp RE, Johnson C, Taylor DN, Wolfson J, Tussey L et al (2011) Safety and immunogenicity of a recombinant M2e-flagellin influenza vaccine (STF2.4xM2e) in healthy adults. Vaccine 29(32):5145–5152 21624416 10.1016/j.vaccine.2011.05.041

[96] Tussey L, Strout C, Davis M, Johnson C, Lucksinger G, Umlauf S et al (2016) Phase 1 safety and immunogenicity study of a quadrivalent seasonal flu vaccine comprising recombinant hemagglutinin-flagellin fusion proteins. Open Forum Infect Dis 3(1):ofw015 26925433 10.1093/ofid/ofw015 PMC4766387

[97] Klimek L, Kundig T, Kramer MF, Guethoff S, Jensen-Jarolim E, Schmidt-Weber CB et al (2018) Virus-like particles (VLPs) in prophylaxis and immunotherapy of allergic diseases. Allergo J Int 27(8):245–255 30546996 10.1007/s40629-018-0074-y PMC6267129

[98] Kayraklioglu N, Horuluoglu B, Klinman DM (2021) CpG oligonucleotides as vaccine adjuvants. Methods Mol Biol 2197:51–85 32827132 10.1007/978-1-0716-0872-2_4

[99] Salem AK, Weiner GJ (2009) Cpg oligonucleotides as immunotherapeutic adjuvants: innovative applications and delivery strategies. Adv Drug Deliv Rev 61(3):193–194 19166888 10.1016/j.addr.2008.12.003 PMC2667912

[100] Zhang Z, Kuo JC, Yao S, Zhang C, Khan H, Lee RJ (2021) Cpg oligodeoxynucleotides for anticancer monotherapy from preclinical stages to clinical trials. Pharmaceutics. 10.3390/pharmaceutics14010073 10.3390/pharmaceutics14010073 PMC8780291 35056969

[101] O’Hagan DT, van der Most R, Lodaya RN, Coccia M, Lofano G (2021) “World in motion”: emulsion adjuvants rising to meet the pandemic Challenges. NPJ Vaccines 6(1):158 34934069 10.1038/s41541-021-00418-0 PMC8692316

[102] Huang Z, Gong H, Sun Q, Yang J, Yan X, Xu F (2024) Research progress on emulsion vaccine adjuvants. Heliyon 10(3):e24662 38317888 10.1016/j.heliyon.2024.e24662 PMC10839794

[103] Li Z, Zhao Y, Li Y, Chen X (2021) Adjuvantation of influenza vaccines to induce cross-protective immunity. Vaccines. 10.3390/vaccines9020075 33494477 10.3390/vaccines9020075 PMC7911902

[104] Vulliet R (1996) Improved technique for the preparation of water-in-oil emulsions containing protein antigens. Biotechniques 20(5):797–800 8723921 10.2144/96205bm14

[105] Cohet C, van der Most R, Bauchau V, Bekkat-Berkani R, Doherty TM, Schuind A et al (2019) Safety of AS03-adjuvanted influenza vaccines: a review of the evidence. Vaccine 37(23):3006–3021 31031030 10.1016/j.vaccine.2019.04.048

[106] Murchu EO, Comber L, Jordan K, Hawkshaw S, Marshall L, O’Neill M et al (2023) Systematic review of the efficacy, effectiveness, and safety of MF59® adjuvanted seasonal influenza vaccines for the prevention of laboratory-confirmed influenza in individuals ≥ 18 years of age. Rev Med Virol 33(3):e2329 35142401 10.1002/rmv.2329

[107] Goksoyr L, Funch AB, Okholm AK, Theander TG, de Jongh WA, Bonefeld CM, & Sander AF (2022) Preclinical efficacy of a capsid virus-like particle-based vaccine targeting il-1beta for treatment of allergic contact dermatitis. Vaccines (Basel) 10(5):828 10.3390/vaccines10050828 PMC9143278 35632584

[108] Janković VV, Velebit B, Lakićević B, Mitrović R, Milojević L (2023) Major allergens – the big nine. Meat Technol 64(2):111–115

[109] Pfaar O, Bousquet J, Durham SR, Kleine-Tebbe J, Larche M, Roberts G et al (2022) One hundred and ten years of allergen immunotherapy: a journey from empiric observation to evidence. Allergy 77(2):454–468 34315190 10.1111/all.15023

[110] Bonertz A, Roberts GC, Hoefnagel M, Timon M, Slater JE, Rabin RL et al (2018) Challenges in the implementation of EAACI guidelines on allergen immunotherapy: a global perspective on the regulation of allergen products. Allergy 73(1):64–76 28771830 10.1111/all.13266

[111] Paoletti G, Di Bona D, Chu DK, Firinu D, Heffler E, Agache I et al (2021) Allergen immunotherapy: the growing role of observational and randomized trial “real-world evidence.” Allergy 76(9):2663–2672 33583050 10.1111/all.14773

[112] Klein NP, Lewis N, Goddard K, Fireman B, Zerbo O, Hanson KE et al (2021) Surveillance for adverse events after COVID-19 mRNA vaccination. JAMA 326(14):1390–1399 34477808 10.1001/jama.2021.15072 PMC8511971

[113] Pan Y, Han Y, Zhou C, Zhao L, Zheng J, Ye X et al (2025) Evaluating the safety of XBB.1.5-containing COVID-19 mRNA vaccines asing a self-controlled case series study. Nat Commun 16(1):6514 40664645 10.1038/s41467-025-61613-4 PMC12264068

[114] McSweeney MD, Mohan M, Commins SP, Lai SK (2021) Anaphylaxis to Pfizer/BioNTech mRNA COVID-19 vaccine in a patient with clinically confirmed PEG allergy. Front Allergy 2:715844 35387046 10.3389/falgy.2021.715844 PMC8974707

